# Effect of UV LED and Pulsed Light Treatments on Polyphenol Oxidase Activity and *Escherichia coli* Inactivation in Apple Juice

**DOI:** 10.1021/acs.jafc.3c08888

**Published:** 2024-06-14

**Authors:** Christelle Pihen, Aurelio López-Malo, Nelly Ramírez-Corona

**Affiliations:** Departamento de Ingeniería Química, Alimentos y Ambiental, Universidad de las Américas Puebla, ExHda Santa Catarina Mártir s/n, San Andrés Cholula, Puebla 72810, México

**Keywords:** pulsed light, ultraviolet light-emitting diodes, enzyme inhibition, bioactive compounds, *Escherichia coli*

## Abstract

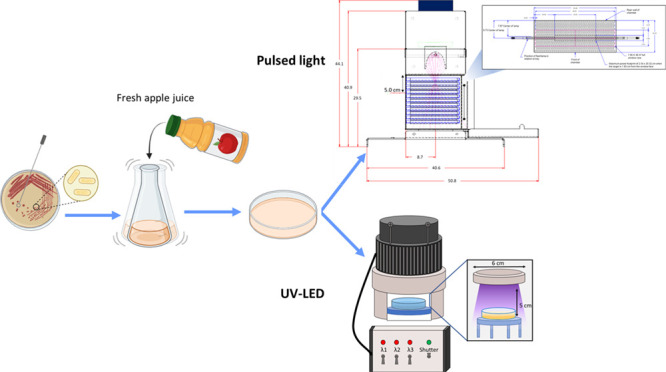

Enzymatic browning in fruits and vegetables, driven by polyphenol oxidase (PPO) activity, results in color changes and loss of bioactive compounds. Emerging technologies are being explored to prevent this browning and ensure microbial safety in foods. This study assessed the effectiveness of pulsed light (PL) and ultraviolet light-emitting diodes (UV-LED) in inhibiting PPO and inactivating *Escherichia coli* ATTC 25922 in fresh apple juice (*Malus domestica* var. Red Delicious). Both treatments’ effects on juice quality, including bioactive compounds, color changes, and microbial inactivation, were examined. At similar doses, PL-treated samples (126 J/cm^2^) showed higher 2,2- diphenyl-1-picrylhydrazyl inhibition (9.5%) compared to UV-LED-treated samples (132 J/cm^2^), which showed 1.06%. For microbial inactivation, UV-LED achieved greater *E. coli* reduction (>3 log cycles) and less ascorbic acid degradation (9.4% ± 0.05) than PL. However, increasing PL doses to 176 J/cm^2^ resulted in more than 5 log cycles reduction of *E. coli*, showing a synergistic effect with the final temperature reached (55 °C). The Weibull model analyzed survival curves to evaluate inactivation kinetics. UV-LED was superior in preserving thermosensitive compounds, while PL excelled in deactivating more PPO and achieving maximal microbial inactivation more quickly.

## 1 Introduction

Apple (*Malus domestica*) is celebrated as one of the most favored fruits, offering various invaluable antioxidants and essential nutrients for human health. In 2023, the leading global producers were China (44.4 million tons), followed by the United States (4.6 million tons), Poland (3.6 million tons), Italy (2.5 million tons), and France (1.8 million tons).^1^ This underscores the significant role of apple production in contributing to countries’ economic growth and development through both exports and local consumption. Moreover, it underscores the nutritional value and diverse bioactive components, including vitamin C and phenols, depending on the variety, that enhance the health benefits associated with apple consumption.^2^

However, the impracticality of storing the substantial volume of harvested fruit necessitates apple processing to avert degradation and spoilage. Among various processing techniques like drying, juicing, jamming, canning, and leathering, juicing stands out as one of the most widely adopted methods for apple preservation. Apple juice, recognized as the second most consumed juice globally, plays a pivotal role in the beverage industry. The leading consumers of apple juice are the United States, Germany, and the United Kingdom, emphasizing its popularity compared to other beverages.^3^

Nonetheless, akin to other beverage production processes, the production and pasteurization of apple juice constitute an energy-intensive food system with adverse effects on both the environment and the final product. Conventional thermal processing methods result in the degradation of heat-sensitive nutrients, including ascorbic acid and phenols, and adversely impact fruit and vegetable juices’ sensory and rheological characteristics.^4^ Consequently, a growing focus in recent decades has been on replacing conventional pasteurization methods, characterized by high temperatures, with more efficient and rapid processes that preserve the bioactive compounds inherent to this product.^5^

Recently, there is a growing interest in using light-based technologies for pasteurization. These technologies include pulsed light (PL) and ultraviolet light-emitting diodes (UV-LEDs). PL is adaptable to utilize a broad spectrum of light, ranging from ultraviolet to infrared (100–1100 nm), and has a short duration for microbial inactivation.^6,7^ During PL treatments, electrical energy is accumulated in capacitors and discharged in high-intensity pulses. The inactivation mechanism of PL is linked to photochemical activity altering DNA through the absorption of the ultraviolet light spectrum. Furthermore, a photothermal^8^ and photophysical^9,10^ effect associated with localized heating of microbial cells can induce cell rupture due to infrared light.^7,9,11^

As an alternative, UV-LEDs are generated using semiconductor materials, enabling emission at various wavelengths.^12^ The color of the emitted light is determined by the band gap energy of the semiconductor material.^13^ Moreover, combining different UV-LEDs that emit light at distinct wavelengths is feasible.^14^ While UV-LEDs have been extensively studied for water treatment, there is limited research regarding their application for fruit juice pasteurization.^14−16^ Further research on this technology must assess its suitability for juice pasteurization and explore its impact on juice quality attributes and bioactive compounds. Comparative studies with other emerging technologies based on broad-spectrum light emission are needed.

Hence, this study aims to compare two light-based emerging technologies—PL and UV-LED—by assessing their impact on the inhibition of the enzyme polyphenol oxidase (PPO), the preservation of bioactive compounds, and the color alteration in fresh apple juice. The microbial inactivation efficiency of UV-LEDs and PL was also examined by inoculating *Escherichia coli* ATTC 25922 in apple juice, and the resulting inactivation kinetics were analyzed using the Weibull model.

## 2 Materials and Methods

### 2.1 Preparation of Fresh Apple Juice

Apples (*Malus domestica* var. Red delicious) were purchased at the local market in San Pedro Cholula Puebla, Puebla, Mexico. Apples were washed, sliced, and processed with a juice extractor (E0802, Turmix), then filtered in a strainer and subsequently centrifuged at 9500 × *g* for 15 min at 4 °C (Beckman Coulter Ltd., Palo Alto, CA, USA). The supernatant was collected and subsequently treated with PL and UV-LED.

### 2.2 Characterization of Apple Juice

The physicochemical properties of apple juice were analyzed before and after UV-LED and PL treatments. Soluble solid content (°Brix) was measured by an ATC-1E refractometer (Atago, Japan). pH was measured with a potentiometer Orion Star A211 (Thermo Scientific, Japan). Viscosity values were determined following the methodology of Gouma et al.^17^ and Müller et al.,^18^ using DV-II + Viscosimeter (Brookfield, Canada). Turbidity (NTU) was determined by Pihen et al.^13^ procedure with a turbidimeter HACH 2100Q (Iowa, United States).

### 2.3 Color Measurement

The color of apple juice was determined before and after UV-LED and PL treatment by Konica Minolta CR 400 Chromameter (Konica Inc., Japan) in Hunter L* (brightness-darkness), a* (redness-greenness), and b*(yellowness- blueness) color scales.^19^ The following equations (eqs 1 and 2) calculated the total color change (Δ*E*) and the browning index (BI) of the samples.
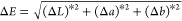
1
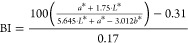
2

### 2.4 PL and UV-LED Treatments

**Figure 1:**
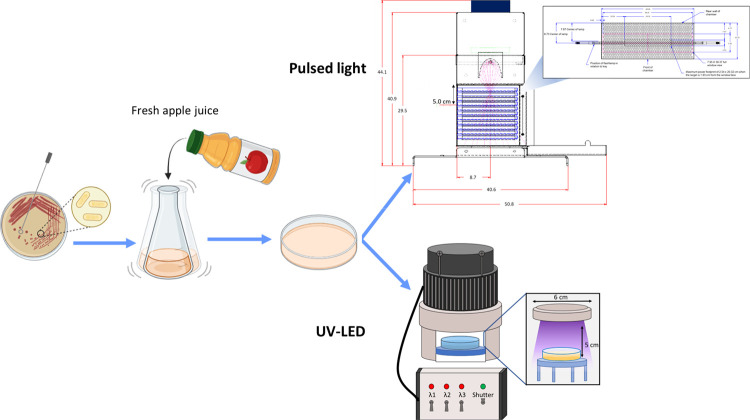
Schematic diagram of PL and UV-LED treatment. The image was created with the assistance of BioRender.

Apple juice underwent PL treatment utilizing the SteriPulse Z-1000 system (XENON Corp., Wilmington, MA). This system featured a linear xenon flash lamp (Ø 2.54 × 40.6 cm, model LH-840, B-type, mercury-free), capable of delivering high-intensity noncollimated white light spanning the range of 100–1100 nm (Figure 1). The lamp produced 2.52 J/cm^2^ per pulse on the strobe surface at an input voltage of 3 kV, with three pulses per second and a pulse duration of 360 μs. During each PL treatment session, inoculated juice was spread in a thin layer (0.5 cm) within a Petri dish (100 × 15 mm), positioned at a perpendicular distance of 5 cm from the lamp surface. The duration of time treatments ranged from 0 to 70 s.

A multiwavelength UV-LED system (PearlLab Beam, Aquisense Technologies, USA) was employed for experimentation. This UV-LED system comprised three channels, each emitting near-monochromatic wavelengths (3 × 255 nm, 3 × 265 nm, 3 × 280 nm), as illustrated in Figure 1. In the UV-LED treatment, the apple juice sample, with a thickness of 0.5 cm, was placed in a Petri dish (60 × 15 mm). The distance between the UV-LED source and the sample was consistently set at 5 cm for all experiments. Treatment involved a combination of wavelengths administered for 20, 40, and 60 min, maintaining an initial temperature of 25 °C.

For both treatments, fluence was computed using data provided by equipment suppliers and equations outlined by Pihen et al.^13^ for fluence determination, taking into account the sample’s thickness and absorbance. The sample temperature was monitored using a thermocouple (SDL200 Extech Instruments, United States) positioned at the center of each Petri dish, at a depth of 0.5 cm for each sample.

### 2.5 PPO Activity

PPO activity was assessed using a spectrophotometric method by Bi et al.^20^ The method relies on measuring the absorbance of brown polymers formed during the oxidation of catechol in the presence of PPO, observed at 410 nm. Catechol served as the substrate, and a 0.05 M catechol solution was prepared in 0.2 M phosphate buffer (pH 6.5). 0.5 mL of the centrifuged juice was added to 2.5 mL of the substrate solution for analysis. The increase in absorbance at 410 nm over 10 min was promptly monitored after a 3-min incubation using a UV-1800 spectrophotometer (Shimadzu Co. Ltd., Japan). The decrease in PPO activity was calculated using eq 3:
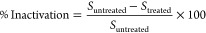
3where *S*_untreated_ and *S*_treated_ represent enzymatic activity in freshly extracted (untreated) juice and UV-LED or PL-treated juice.

### 2.6 Phytochemical Properties of Apple Juice

The total phenolic content of treated and untreated samples was determined using the Folin–Ciocalteu method described by Unluturk et al.^19^ A calibration curve of Gallic acid was prepared. Sample concentrations were determined from the calibration curve (*y* = 1249.4*x* – 14.466) and expressed as mg of Gallic acid equivalents per milliliter of sample, GAE/mL. Juice samples were diluted in distilled water and mixed with 2.5 mL of Folin-Ciocalteu phenol reagent followed by 2 mL of Na_2_Co_3_ addition. The reactive mixture was allowed to stand for 3 h in darkness and was quantified at 740 nm using a UV–vis spectrum UV-1900i (Shimadzu, Kyoto, Japan).

Total antioxidant activity was evaluated using the colorimetric method based on free radical scavenging sample capacity, using 2,2- diphenyl-1-picrylhydrazyl (DPPH) stable radical according to the methodology proposed by Bi et al.^20^ The DPPH method works by the antioxidant’s reduction of the violet DPPH radical via a hydrogen atom transfer mechanism, resulting in a color change to stable pale-yellow. This method is used to measure the ability of various compounds (some vitamins, phenolic compounds, flavonoids, anthocyanins, etc.) to act as free radical scavengers, being useful to evaluate the antioxidant activity of beverages. The remaining violet DPPH radical is measured employing a UV–vis spectrophotometer at approximately 515–520 nm.^21^ The apple juice (0.1 mL) was mixed with 4.0 mL of DPPH solution (0.14 mmol/L in methanol). The mixture was shaken and kept in the dark for 45 min at room temperature. The mixture of 0.1 mL of methanol and 4.0 mL of DPPH was used as a control. The absorbance at 517 nm of all mixtures was measured by UV–vis spectrum UV-1900i (Shimadzu, Kyoto, Japan). The percentage quenching of the DPPH radical was calculated based on the observed decrease in the absorbance. The radical scavenging activity was calculated using eq 3. The calibration curve between %Inhibition and known solutions of Trolox was then established. The radical scavenging activities of the test samples were expressed as Trolox equivalent antioxidant capacity (μM TE/g) on their percentage inhibitions. Trolox standard solutions were prepared at a concentration ranging from 100 to 2000 μM of Trolox.

The analysis of ascorbic acid in apple juices was conducted before and after subjecting them to UV-LED and PL treatments, utilizing the official method 967.21 AOAC (2000).

### 2.7 *Escherichia coli* Inoculation and Enumeration

*Escherichia coli* ATCC 25922, chosen as a surrogate for pathogenic *Escherichia coli*,^22^ was selected due to its association with numerous food intoxication outbreaks and its role as a control bacterium in juice.^23,24^ This strain of *Escherichia coli* ATCC 25922 was sourced from the bacterial collection at the Food Microbiology Laboratory of the Universidad de las Americas Puebla. Bacteria were grown in 50 mL of sterile trypticase soy broth (BD Bioxon, Estado de México, México) at 37 °C for 18 h for each test and then inoculated in 25 mL for each pasteurized food model solution, corresponding to 0.5 cm of thickness in Petri dish of each sample evaluated, by sterilization at 120 °C for 15 min, with an inoculum around 10^7^ CFU/mL.

After the treatment, apple juice samples were serially diluted in sterile saline, surface plated on trypticase in soy agar, incubated for 24 h at 37 °C, and finally, colony counted.

### 2.8 Weibull Model

Even though various nonlinear mathematical models are available, the Weibull model was chosen for its consistent performance in previous studies involving light-based technologies for fruit derivative processing.^22,25,26^ Additionally, several research studies on emerging technologies, specifically PL and UV-LED treatment, have employed the Weibull model to predict microbial inactivation kinetics.^25,27,28^ The Weibull model (eq 4) described the PL and UV-LED inactivation kinetics for *E. coli* ATCC 25922.
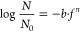
4where *N* is the number of survivors after the PL treatment, *N*_0_ is the initial inoculum level, *b* is the scale parameter, *n* is the shape parameter, and *f* is the fluence (J/cm^2^). The shape parameter indicates the shape of the survivor curve, with *n* > 1 indicating concave-down survival curves, *n* < 1 concave-up survival curves, and *n* = 1 indicating linear survival curves.

The values of *b* and *n* were used to generate the frequency distributions of resistance by eq 5
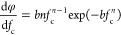
5where *f*_c_ is a measure of the organism’s resistance or sensitivity, and  is the Weibull distribution corresponding to *f*_c_. Additional statistical parameters as mode, mean, variance, and coefficient of skewness were also calculated.

### 2.9 Statistical Analysis

All the experiments were performed in triplicate. The experimental data were evaluated using Minitab 18 (Minitab Inc., State College, PA, ABD) for the experimental design and the analysis of variance (ANOVA). Tukey’s pairwise comparison test was used to compare the means of data at a 95% confidence interval (*p* < 0.05).

## 3 Results and Discussion

### 3.1 Effect of PL and UV-LED Treatments on the Physicochemical Properties of Apple Juice

Table 1 displays the differences observed in fresh apple juice treated with PL and UV-LED at a similar fluence. The soluble solids (°Brix) and viscosity of the samples remain consistent with the control sample (14.40°Brix and 13.96 cP) despite the PL and UV-LED treatment. Additionally, there was no significant difference in water activity (*a*_w_) after both treatments in comparison to the untreated sample. However, a significant difference in the turbidity of the samples treated with PL (53.0 ± 1.03 NTU) and UV-LED (63.3 ± 3.06 NTU) was observed.

**Table 1:** Physicochemical Properties of Fresh Apple Juice

	**pH**	**°Brix**	**viscosity (cP)**	**turbidity (NTU)**	**α(cm**^**–1**^**)**	***a***_**w**_
no treatment	3.48 ± 0.01^a^	14.40 ± 0.05^a^	13.96 ± 0.15^a^	81.3 ± 3.21^a^	7.44 ± 0.05 ^a^	0.984 ± 0.01^a^
PL (126 J/cm^2^)	3.47 ± 0.01^a,b^	14.20 ± 0.14^a^	13.92 ± 0.23^a^	53.0 ± 1.03^b^	7.37 ± 0.02 ^a^	0.987 ± 0.01^a^
UV-LED (132 J/cm^2^)	3.44 ± 0.01^b^	14.43 ± 0.11^a^	13.93 ± 0.79^a^	63.3 ± 3.06^c^	7.53 ± 0.04 ^a^	0.989 ± 0.01^a^

The results obtained for fresh apple juice regarding physicochemical properties like pH and soluble solids resemble those found by Akgün & Ünlütürk^14^ for carrot, carob, ginger, grape, and lemon treatments, Koutchma^29^ for apple juice, and Baykus^19^ for a mixed beverage treated with UV-LED, as well as Chakraborty et al.^30^ for apple, carambola, and grape juice treated with PL. For both treatments, no significant changes were observed between treated and untreated samples. Nonetheless, there is a void in the literature regarding changes in viscosity and turbidity, pre- and post-UV-LED, and PL treatments in fresh apple juice. In this regard, Pierscianowski et al.^31^ identified notable differences in the viscosity of kale juice subjected to UV-LED treatment. As a first assumption, differences in turbidity may be related to changes in color and temperature during treatment and the possible degradation of certain compounds during enzymatic and microbial inactivation induced by light. Moreover, Table 2 displays the variations in color parameters on CIELab space (L*, a*, and b*), along with the color change and temperature for each treatment (PL and UV-LEDs). In the case of the PL treatment, the primary shifts in color were noted in the a* and b* parameters of the Hunter scale, representing changes in redness-greenness and yellowness-blueness, respectively. In both instances, these values exhibited a notable decrease with higher treatment doses. On the other hand, with UV-LED treatment, the L* parameter associated with brightness-darkness and the b* parameter decreased with increasing doses, whereas the a* parameter showed a significant increase as the delivered dose to the sample rose. Therefore, it was evident that the samples subjected to UV-LEDs were significantly affected with a more pronounced color change and browning index compared to the control sample and the samples treated with PL, particularly after 40 and 60 min of treatment, with an Δ*E* of 4.36 and 5.08, respectively. This change is probably caused by the oxidation of certain phytochemicals present in apple juice samples during a long exposure time by UV-LED.^19^

**Table 2:** Color and PPO Inactivation by PL and UV-LED in Apple Juice

	**time (s)**	**dose** (J/cm^2^)	L*	a*	b*	**Δ***E*	**BI/BI**_**0**_	**temperature (°C)**	**%inactivation PPO**	***R***^**2**^
control			42.59 ± 0.51^a^	3.77 ± 0.35^a^	15.36 ± 1.76^a^			22.7 ± 0.8		
PL	30	75.7	40.59 ± 0.98^b^	4.47 ± 0.50^c^	14.61 ± 2.06^a,b^	2.25 ± 1.31^a^	1.01 ± 0.01^b,c^	39.5 ± 0.5	15.0	0.98
	50	126.1	40.52 ± 1.77^b^	4.24 ± 0.48^a,c^	14.64 ± 1.71 ^a,b^	2.24 ± 0.65^a^	1.01 ± 0.01 ^b,c^	43.9 ± 0.4	18.7	0.97
	70	176.6	40.38 ± 0.90^b^	2.39 ± 0.23^d^	12.36 ± 1.02^c^	3.97 ± 1.11^b^	0.96 ± 0.02^e^	55.7 ± 0.9	27.7	0.97
UV-LED	1200	44.1	42.32 ± 0.03^a^	4.59 ± 0.06^c^	17.63 ± 0.16^d^	2.43 ± 1.73^a^	1.04 ± 0.01^a^	22.7 ± 0.3	2.2	0.96
	2400	88.1	39.53 ± 0.05^b,c^	4.74 ± 0.03^c,e^	12.41 ± 0.06^c^	4.36 ± 1.24^c^	0.98 ± 0.01^d^	22.1 ± 0.4	11.2	0.99
	3600	132.2	38.36 ± 0.04^c^	5.25 ± 0.02^b,e^	12.97 ± 0.02^c,b^	5.08 ± 0.88^d^	1.00 ± 0.01^c,d^	22.1 ± 0.2	18.0	0.99

On the other hand, color changes observed in apple juice treated with PL align with the results reported by Palgan et al.^32^ They noted a noteworthy shift in the a* parameter of the Hunter scale when the treatment time increased from 2 to 8 s, with a total energy of 28 J/cm^2^, similar to the apple juice browning index reported by Ferrario et al.,^33^ which is approximately unity, as shown in our study (Table 2). Nevertheless, the color shift for UV-LED surpasses that reported by Baykus et al.,^19^ despite their use of LED light exclusively at wavelengths of 280 and 365 nm. Conversely, the BI and Δ*E* values elevation may be attributed to residual enzyme activities. Akgün and Unlütürk^14^ highlighted the presence of residual PPO activity in apple juice samples after UV-LED treatments, suggesting that this contributed to alterations in browning and, consequently, to color changes and browning index.

It is worth noting that PL treatment can cause a significant increase in temperature, even for short periods of radiation. In the case of the most intense PL treatments (177 J/cm^2^), temperature rose from 22.7 to 56 °C. Therefore, the effectiveness of inactivation should be evaluated based on the combined effects of both photochemical and photothermal mechanisms. On the other hand, for UV-LED treatment, the temperature of the samples remains relatively stable even with extended processing times.

### 3.2 Inhibition of PPO Enzyme Activity by PL and UV-LED

The oxidoreductases, such as PPO enzymes, present in apple juice are responsible for undesirable browning and color change. Table 2 illustrates the variations in PPO activity in fresh apple juice subjected to different doses of UV-LED and PL treatments. The most substantial PPO inactivation rates were recorded at 27.7 and 17.95% for fluences of 176.6 J/cm^2^ with PL and 132.2 J/cm^2^ with UV-LED, respectively. Although the most effective PPO inactivation occurred at 176.6 J/cm^2^ with PL treatment, it is noteworthy that a dose of 126.1 J/cm^2^, applied during a 50-s PL treatment, also resulted in a notable 18.7% PPO inactivation. This inhibition activity is comparable to the highest UV-LED dose evaluated, which achieves an 18% PPO inhibition but requires a longer treatment time of 60 min.

Nevertheless, it is crucial to highlight that the efficacy of enzyme inactivation through light treatments, particularly in the UV range, is contingent on the juice matrix and its composition, as elucidated by Müller et al.^34^ and Akgün and Ünlütürk.^14^ The considerable absorption coefficient (α = 7.44 cm^–1^, Table 1) of apple juice suggests that the penetration depth of UV–C light into the juice is constrained, resulting in a diminished level of enzyme inactivation. Chakraborty et al.^30^ noted a more pronounced decline in PPO activity in gooseberry juice subjected to PL treatment as the power intensity increased. Meanwhile, Akün and Ünlütürk^14^ examined the inactivation of PPO in cloudy apple juice treated for 40 min by UV-LEDs at individual and combined wavelengths (254/280/365/405 nm). They found the highest residual PPO activity at 254 nm (70.4% PPO residual) when applied individually, and this trend persisted when combinations that included the 254 nm wavelength were employed. It is important to note that the inactivation percentage increases as more wavelengths and doses are combined. This observation correlates with the browning results (BI/BI_0_), with increasing dosage in both devices reducing the samples’ darkening in Table 2.

Nevertheless, due to the partial resistance of the browning enzyme to light treatments, the activity of PPO and the intermediate and end products of the Maillard reaction resulted in a noteworthy alteration in the color parameters Baykus et al.^19^ As a result, the degradation of pigments in the apple juice samples subjected to UV-LED treatment is more pronounced compared to PL treatment, as the latter did not achieve significant inhibition of PPO.

### 3.3 Effect of UV-LED and PL Treatments on the Phytochemical Attributes of Apple Juice

**Figure 2:**
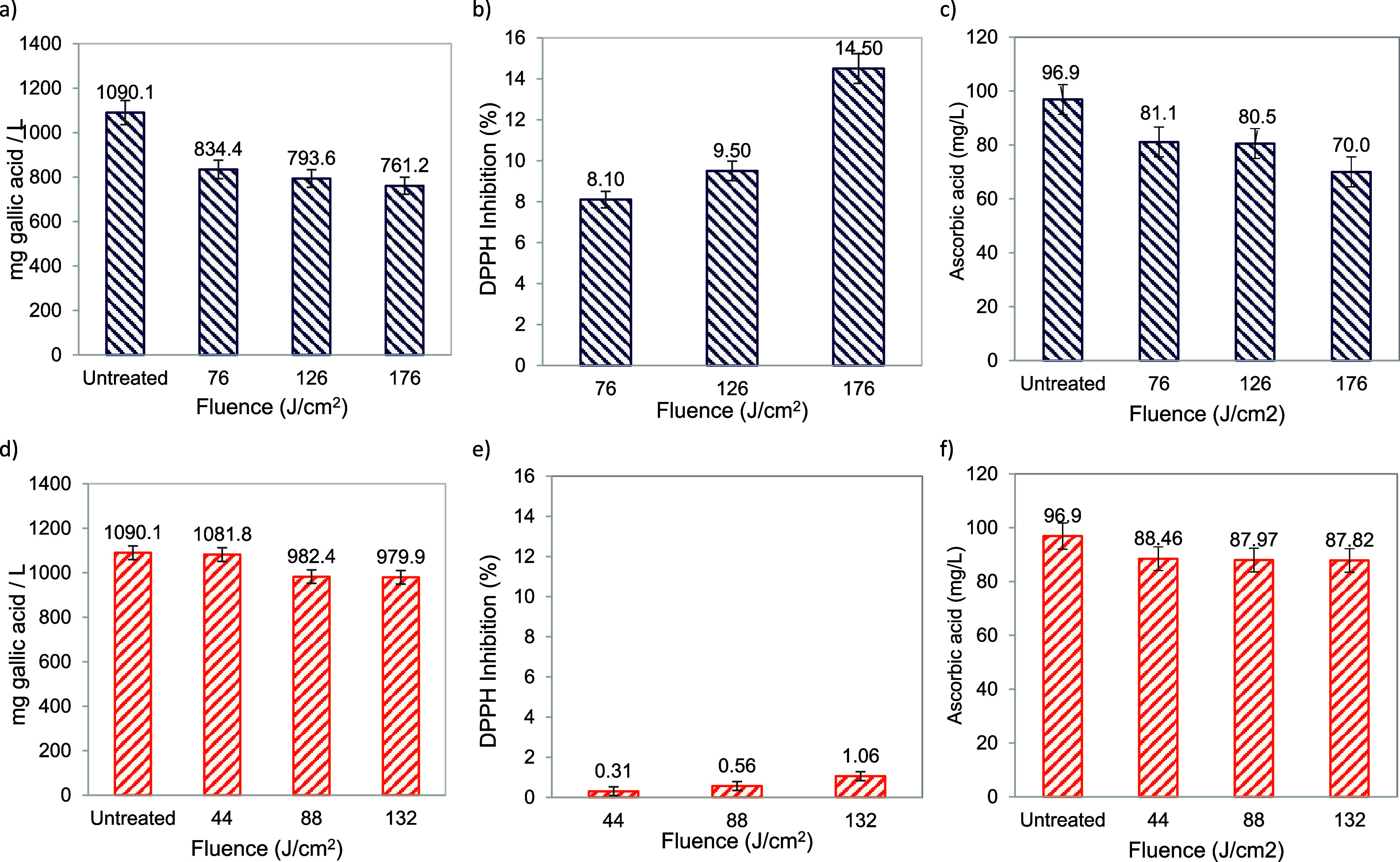
Changes in total phenolic compounds ((a) PL and (d) UV_LED), total antioxidant capacity ((b) PL and (e) UV_LED), and vitamin C ((c) PL and (f) UV_LED) in apple juice.

Figure 2 shows the change in total phenols (Figure 2a,d), antioxidant capacity (Figure 2b,e), and vitamin C (Figure 2c,f) in fresh apple juice after treatments with PL and UV-LED. Notably, the PL treatment induces a significant reduction in total phenols (Figure 2a), antioxidant activity (Figure 2b), and vitamin C (Figure 2c) in apple juice compared to UV-LED treatment. At the maximum treatment of 176.4 J/cm^2^ with a short duration (70 s), there is a decrease of 38.4% in total phenols, 14.5% in DPPH inhibition, and 38.4% in vitamin C (70 mg/L ascorbic acid) in the fresh apple juice samples.

Despite the brief treatment time, the remarkable reduction in these compounds can be attributed to the dual treatment mechanism of the PL equipment, involving photochemical and photothermal effects. The latter is evident by the increase in temperature of the PL-treated samples to approximately 60 °C (Table 1), leading to a higher loss of bioactive compounds. However, this temperature increase aids in the inactivation of enzymes associated with browning (Table 2).

However, it is important to emphasize that, although the inactivation of PPO by UV-LED is not as extensive as that achieved by PL treatment (Table 2), UV-LED treatment at 132.2 J/cm^2^ exhibits lower losses of bioactive compounds compared to untreated samples, with a 9.4% reduction in vitamin C (87.82 mg ascorbic acid/L) and a 10.1% reduction in total phenols (979.9 mg gallic acid/L).

Moreover, at a similar dose exposure using both technologies, 126 J/cm^2^ by PL and 132 J/cm^2^ by UV-LED, the loss of bioactive compounds (total phenolic compounds, total antioxidant capacity, and vitamin C) by PL remains higher to UV-LED (Figure 2). A substantial concentration of total phenols may suggest the presence of a significant substrate reservoir for the PPO enzyme in the enzymatic browning reaction. This, in turn, could produce a substantial quantity of browning product, namely melanin.^19,35^

Furthermore, the initial concentrations of ascorbic acid and total phenols align with the values documented by Szczepańska et al.,^36^ indicating a content of 131.41 mg/L of vitamin C and 1006.7 mg GAE/L for total phenol content. Nevertheless, Unluturk^19^ observes an increase in total phenols in a juice blend of carrot, carob, ginger, grape, and lemon juice following UV-LED treatment (280 and 365 nm) compared to the control sample. Xiang et al.^15^ demonstrated analogous trends in apple juice subjected to UV-LED treatment at 275 nm with a maximum fluence of 1200 mJ/cm^2^, revealing minimal reductions in the total phenol content, antioxidant compounds, and color alteration.

In the context of PL treatment, Chakraborty et al.^30^ noted a diminished loss of antioxidant capacity (6.6%), phenolic compounds (14.5%), and vitamin C (16.3%) with a treatment intensity of 1804 J/cm^2^ in Indian gooseberry juice. Such results align with the reductions in bioactive compounds and antioxidant capacity documented by Chakraborty et al.^37^ for a blend of apple and carambola juice treated at 5000 J/cm^2^, with the levels of total phenols and ascorbic acid being similar to those obtained in our study after PL treatment. Nevertheless, Bhagat and Chakraborty^38^ did not witness substantial changes in total phenols, antioxidant capacity, and vitamin content levels in pomegranate juice when subjected to a maximum dose of 2988 J/cm^2^ using PL. Additionally, they noted a minimal change in the browning index of the pomegranate juice during the different treatment times and doses per PL.

Hence, while PL treatment exhibits a more pronounced impact on bioactive compounds like vitamin C, leading to a more significant inhibition of antioxidant activity, the noteworthy reduction in phenol content prevents a substantial alteration in color and browning rate compared to UV-LED. This is attributed to the limited substrate for the PPO enzyme in the enzymatic browning reaction, minimizing the generation of a significant quantity of browning products.

### 3.4 *Escherichia coli* Inactivation by PL and UV-LED

**Figure 3:**
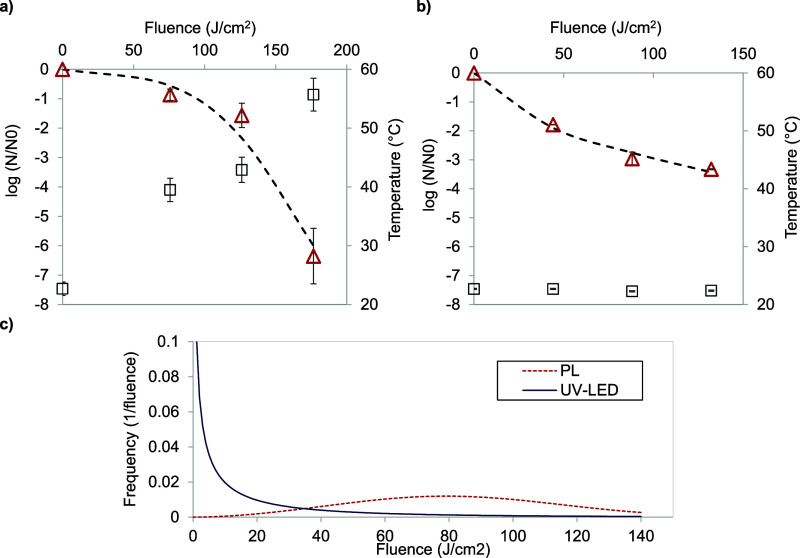
PL (a), UV-LED (b) survival curves of *Escherichia coli* ATTC 25922; triangles indicate experimental data, lines represent the Weibull model estimation, and squares represent the final temperature in each treatment. (c) Frequency distribution of resistances for survival curves by both PL and UV-LED treatments.

The inactivation of *Escherichia coli* ATCC 25922 in apple juice through UV-LED and PL systems, alongside examining the temperature variations throughout the treatments, as illustrated in Figure 3. The Weibull model fitted inactivation kinetics for both cases (see Table 3). Treatment with PL for 70 s reduced 6.35 log CFU/mL of *E. coli* ATCC 25922 at a dose of 176 J/cm^2^. In comparison, UV-LED treatment achieved a lower reduction at a maximum dose of 132 J/cm^2^ with a treatment time of 60 min. However, at similar doses between PL and UV-LEDs (126 and 132 J/cm^2^, respectively), UV-LEDs demonstrated a higher reduction of 3.42 log CFU/mL of *E. coli* compared to PL, resulting in an inactivation of 1.56 log CFU/mL of *E. coli* ATCC 25922. Notably, the time difference between both reductions is significant, with PL requiring 50 s and UV-LEDs necessitating 2400 s.

**Table 3:** Weibull Model Inactivation Parameters for Apple Juice Treated by PL and UV-LEDs

**treatment**	**PL**	**UV-LED**
*b*(J/cm^2^)	3.11 × 10^–6^	0.25
*n*	2.8	0.54
*R*^2^	0.98	0.99
mode	79.13	
mean	82.51	22.83
variance	1017.50	2112.52
skewness coefficient	0.82	6.00

As discussed before, PL treatment causes a significant temperature rise, reaching 55 °C during the 70-s treatment period. While the temperature may not reach levels high enough to reduce microbial growth by itself,^39−41^ it is sufficient to induce a synergistic effect between the PL inactivation mechanism and the temperature increase. The inactivation kinetics for both treatments demonstrated a fit with *R*^2^ > 0.95 (Table 3). The scale parameter (*b*), indicating the inactivation efficiency of *E. coli*, showed higher efficiency with PL compared to UV-LED. The shape parameter (*n*) revealed that if *n* is less than 1 (concave curves), the remaining cells become more resistant to radiation treatment, as observed in Figure 3a. This implies an adaptive capacity to the applied treatment, potentially elucidating why, at a fluence of 126 J/cm^2^ with PL, *E. coli* ATCC 25922 shows increased resistance to inactivation. Conversely, if *n* is greater than 1 (convex curves), it indicates an accumulation of the lethal effect, as shown in Figure 3b, leading to an increase in the rate of destruction with the cumulative form of the Weibull distribution.^42^ The frequency distributions, generated according to eq 5, are shown in Figure 3c. Table 3 also includes the statistical parameters of each frequency distribution: mode, mean, variance, and skewness coefficient.

The frequency distribution corresponding to UV-LED treatment did not have a peak and was skewed to the right (with high skewness coefficients and no mode); this behavior indicated that most of the population was inactivated within the fluence range tested. PL treatment exhibited a flat frequency shape with considerable data spread, large mode, and mean and variance values (with a tail). This indicates that an important fraction of the *E. coli* population survived after the treatment, leaving a fraction of more resistant members, which were much less affected.

The results obtained in this study align with those reported by Sauer and Moraru,^22^ at a maximum fluence of 12 J/cm^2^ with PL. They reduced 5-log CFU/mL for both pathogenic and nonpathogenic *E. coli* O157:H7, with a Weibull model fit (R2) exceeding 0.95 for apple juice treated by PL. Additionally, Ferrario and Guerrero^33^ documented analogous parameter values and a Weibull model fit for both cloudy and clarified apple juice when treated with PL at a maximum dose of 0.73 J/cm^2^, leading to an inactivation of <2 log CFU/mL of *E. coli*. Meanwhile, Chakraborty et al.^37^ showcased remarkable efficacy in eliminating molds, yeasts, and mesophilic aerobic bacteria in a blend of juices containing apple, carambola, and grape, treated with a total fluence of 5000 J/cm^2^ by PL.

On the other hand, Baykus et al.^19^ obtained a reduction of 3.44 log CFU/mL of *E. coli* K12, a result comparable to our findings. Such a reduction was achieved using a combination of wavelengths (280/365 nm) at a dose of 173 J/cm^2^ in a mixed beverage comprising a blend of carrot, carob, ginger, grape, and lemon juice. Akgün and Ünlütürk^14^ accomplished a maximum inactivation of 3.5 log CFU/mL of *E. coli* K12 through treatment using four UV-LEDs emitting light at 254 nm, with a dose of 707.2 J/cm^2^. Furthermore, Xiang et al.^15^ illustrated an inactivation of <6 log CFU/mL of *Z. rouxii* in apple juice treated with 64 LEDs at 275 nm configuration, reaching a maximum dose of 1200 mJ/cm^2^. Despite Xiang et al.^15^ achieving a higher level of inactivation, this underscores UV-LEDs’ efficiency in treating bacteria and yeast while simultaneously preserving bioactive compounds of interest to consumers.

Overall, there are clear differences in enzyme inhibition, preservation of phytochemical compounds, and microbial inactivation in apple juice treated with both technologies. These differences arise from the distinct mechanisms associated with each technology. In particular, compared to UV-LED, the increased inactivation observed in PL treatment can be attributed to a synergistic effect induced by the temperature elevation. The use of UV-LED treatment demonstrates to be more efficient in preserving thermosensitive compounds like vitamin C and phenols. Contrarywise, the PL treatment is highly effective in rapidly deactivating a higher percentage of PPO and reducing *E. coli* by over 5 log cycles in apple juice. These findings underscore the versatility of both technologies as an alternative for total or partial replacement of thermal treatments in food pasteurization, especially for heat-sensitive compounds. Future research in this field should explore combined treatments that take advantage of the effectiveness of PL and complement the strengths of UV-LED treatment in ensuring product quality and safety. Conducting sensory studies becomes crucial in helping identify the presence of off-flavors and assess the acceptability of products treated by these technologies. Furthermore, it is relevant to investigate the formation of hydroxymethylfurfural compounds, influenced by the technologies involving light and heat.
